# Testing dataset for head segmentation accuracy for the algorithms in the ‘BGSLibrary’ v3.0.0 developed by Andrews Sobral

**DOI:** 10.1016/j.dib.2020.106385

**Published:** 2020-10-08

**Authors:** Seng Cheong Loke, Bruce A. MacDonald, Matthew Parsons, Burkhard C. Wünsche

**Affiliations:** a Faculty of Medicine and Health Sciences, University of Auckland, New Zealand; b Faculty of Engineering, University of Auckland, New Zealand; c University of Waikato, New Zealand; d Faculty of Science, University of Auckland, New Zealand

**Keywords:** Background initialization, Background maintenance, Background modeling, Background subtraction, Foreground detection, Foreground segmentation, Portrait segmentation

## Abstract

This dataset consists of video files that were created to test the accuracy of background segmentation algorithms contained in the *C*++ wrapper ‘BGSLibrary’ v3.0.0 developed by Andrews Sobral. The comparison is based on segmentation accuracy of the algorithms on a series of indoor color-depth video clips of a single person's head and upper body, each highlighting a common factor that can influence the accuracy of foreground-background segmentation. The algorithms are run on the color image data, while the ‘ground truth’ is semi-automatically extracted from the depth data. The camera chosen for capturing the videos features paired color-depth image sensors, with the color sensor having specifications typical of mobile devices and webcams, which cover most of the use cases for these algorithms. The factors chosen for testing are derived from a literature review accompanying the dataset as being able to influence the efficacy of background segmentation. The assessment criteria for the results were set based on the requirements of common use cases such as gamecasting and mobile communications to allow the readers to make their own judgements on the merits of each algorithm for their own purposes.

## Specifications Table

**Table:** 

Subject	Computer Science - Computer Vision and Pattern Recognition
Specific subject area	Foreground-background segmentation of the head and upper body.
Type of data	Table Figures Video clips
How data were acquired	Intel RealSense Depth Camera D435 featuring a global shutter, large color pixels of 3 um square, and a depth sensor using disparity mapping from stereo infra-red cameras. Custom acquisition software found at GitHub repository https://github.com/scloke/SegTest. Testing was performed on a system using Microsoft Windows 10 with a four-core Intel Xeon E3 processor running at 3.5 GHz using Visual Basic and Visual *C*++ 2019 in a 64-bit address space with 32 GB RAM allocated and an NVIDIA GeForce GTX 1060 6GB graphics card installed. Image processing was done using the libraries in EmguCV 3.2.0 and Accord.Net 3.8.0. The system speed was rated at 475 million floating points per second (MFLOPS) using the Intel processor diagnostic tool 2.10, 64-bit version.
Data format	Raw Analyzed
Parameters for data collection	The following camera settings were used: structured light projector on, autofocus enabled, autoexposure disabled, automatic white balancing disabled, backlight compensation disabled, and powerline frequency compensation disabled. Capture resolution was 640 × 480 pixels at 30 frames per second (fps) for color data and 90 fps for depth data, with the depth data processed using temporal and spatial smoothing with hole-filling to reduce artefacts. Synthetic paired color and depth frames were motion interpolated from the source frames to generate video clips without any inter-frames. The clips were then saved to Audio Video Interleave (AVI) files using FFMPEG, with dimensions of 640 × 480 pixels at 30 fps. Color clips were encoded using the lossy MPEG-4 Part 2 codec at a bit rate of 4 megabit per second (Mbps) except for the noise clips which were encoded at 12 Mbps to preserve the noise artefacts. The clips were captured at night under controlled bidirectional diagonal and side lighting with Philips Hue White & Color Ambiance bulbs set to the ‘Energize’ preset with a color temperature of 6410 K, calculated from the Mired Color Temperature supplied by the Philips Hue Software Development Kit. The camera was placed 120 cm in front of either a plain green screen (standard), a cream-colored screen (camouflage), or with the screen removed (complex), having the subject standing 60 cm in front of the screen, with no intervening objects. This resulted in a foreground area that was consistently about half the total background size, which is sufficiently balanced to not distort the measures of segmentation efficacy, and yet not too big which would prevent the face detection routine from working properly.
Description of data collection	One of the chosen factors listed in Table 2 was then applied. All clips were 40 s long with the first 10 s showing just the background. The subject entered the scene at the 10 s mark and stood in the center of the frame while keeping a neutral expression, with the face and upper body fully visible. The comparison period was set to all frames between the 20 and 40 s mark inclusive. The brightness for all clips was normalized by applying the appropriate constant gamma correction to keep the average pixel brightness throughout the clip at 50% of the maximum brightness. For lighting change factors, the lighting conditions were altered mid-way through the comparison period. For the ‘Ghost Images’ clip, the comparison period was set to one second after the transition. This is because most algorithms for detection and removal of moving objects, ghosts, and shadows operate in less than a second. The comparison period for the ‘Sleeping Foreground’ clip was set to 10 s after the transition to allow enough time for the object to be incorporated into the background model. In clips where a subject was present, the face location was determined using libfacedetection by Shiqi Yu, and a seed point and depth obtained from the center of the bounding rectangle. The ‘ground truth’
	foreground was then extracted from a floating range flood fill starting at the seed point, with a maximum difference of 2 cm between adjacent pixels. Since the depth data from disparity mapping is coarse and lacks edge accuracy, an automated GrabCut algorithm was used to refine the edges of the foreground. The ‘ground truth’ clips were verified by visually inspecting at 4 fps, and adjustments were made with manual GrabCut assistance where the areas of inaccuracy exceeded 5% of the foreground (Fig. 1). For clips without a subject, the depth data was ignored and the ‘ground truth’ foreground was set to zero. The segmentation foreground was obtained by processing the color data using the appropriate ‘BGSLibrary’ algorithm with default settings, except for the ‘GMG’ and ‘GMM KaewTraKulPong’ routines which require an earlier version of OpenCV. To speed up processing, the segmentation routines were run in four isolated parallel threads in ‘release’ mode. The ‘ground truth’ and segmentation clips were saved to AVI files encoded with the lossless FFV1 codec.
Data source location	Institution: University of Auckland City/Town/Region: Auckland Country: New Zealand Latitude and longitude (and GPS coordinates, if possible) for collected samples/data: −36.86, 174.77
Data accessibility	With the article (Figures and Tables) Repository name: Mendeley Data Data identification number: 10.17632/yw5k28z97d.1 Direct URL to data: http://dx.doi.org/10.17632/yw5k28z97d.1 (Video clips)

## Value of the Data

• While there have been many routines developed for foreground-background portrait segmentation, evaluation of these routines is usually done with video clips under standard conditions. Little is known about their performance under some of the factors which affect segmentation efficacy.
• This data will be useful to researchers who are developing and testing algorithms for portrait segmentation.
• Color and ‘ground truth’ video clips are available for each of the factors described. A new algorithm can be tested by applying it to the color video clip and generating the segmented video. This can then be compared against the ‘ground truth’ to obtain any of the metrices of segmentation efficacy.
• Alternatively, the custom software available at https://github.com/scloke/SegTest can be adapted to run the efficacy test automatically, capture new clips, and generate corresponding ‘ground truth’ clips.
• The custom software also contains *C*++ headers which can be used to directly interface OpenCV image structures with the algorithms in BGSLibrary’.

## 1 Data Description

Background segmentation is the process whereby an object of interest in the foreground of an image, often a person, is separated from the background. Typical applications are news and weather casting, game livestreaming, video conferencing and chat software, photo augmentation (Snapchat filters and beauty apps), and technical help desks [1].

Accurate segmentation is quite difficult to achieve, and the method of choice depends on the application and the factors governing the process. In this article, the term ‘factor’ is defined as a characteristic of a particular use case that will influence the efficacy of foreground-background segmentation of an algorithm.

The data consists of a comparison of background segmentation algorithms contained in the *C*++ wrapper ‘BGSLibrary’ v3.0.0 developed by Andrews Sobral [2, 3]. The comparison is based on segmentation efficacy and speed of the algorithms when applied on a series of indoor color-depth video clips of a single person's head and upper body, each highlighting a common factor as determined from the literature review [4]. The algorithms are run on the color image data, while the ‘ground truth’ is semi-automatically extracted from the depth data. The purpose of this article is to provide a reference resource for those who are developing applications that require background segmentation of the head and upper body.

**Fig. 1:**
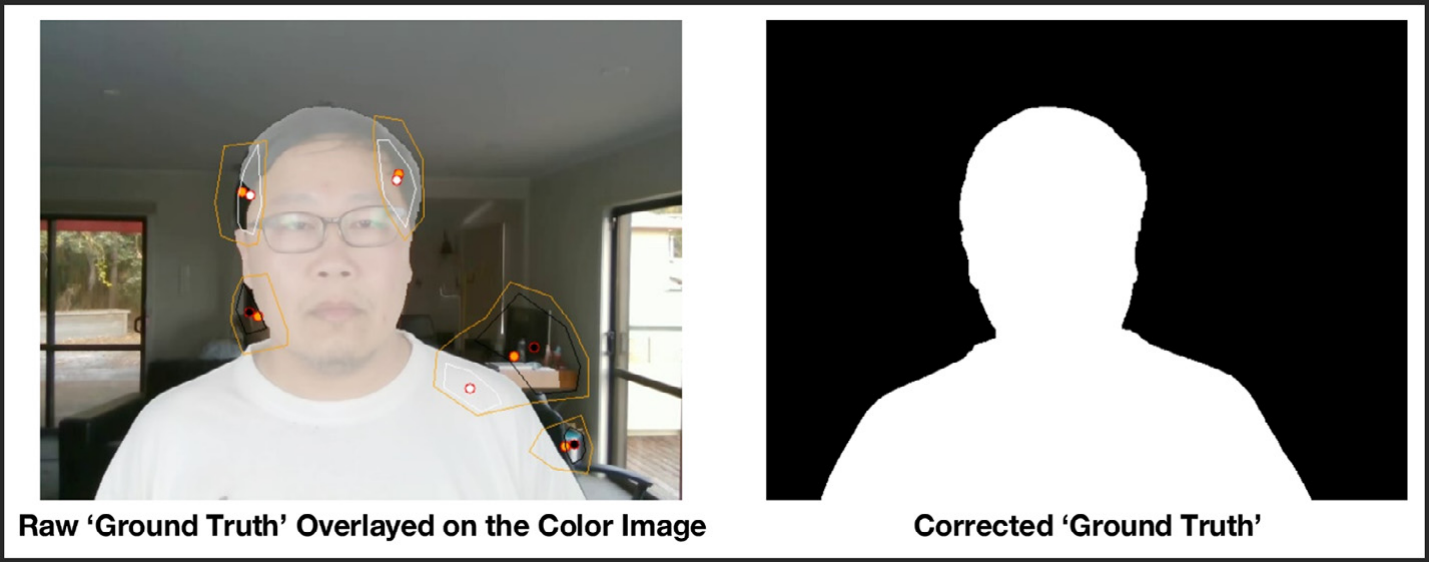
Manual GrabCut correction of raw 'ground truth' images. Note: The raw ‘ground truth’ image on the left shows a filling defect on the right hairline and an incorrectly segmented part of the desk adjacent to the left shoulder. The colored polygons manually mark out areas for the GrabCut algorithm to correct, being region of interest (orange), true foreground (white), and true background (black).

Fig. 1 demonstrates the process whereby the automatic extraction of ‘ground truth’ images is corrected after inspection of the video frames at 4 fps with changes made using a manual GrabCut process. In previous segmentation datasets, the ‘ground truth’ was extracted manually with teams of data processors hand-drawing boundaries between domains [5]. This was not feasible for the present dataset given the thousands of individual frames for the video clips, so a semi-automated process was used to refine the raw ‘ground truth’ images obtained from the depth camera.

**Fig. 2:**
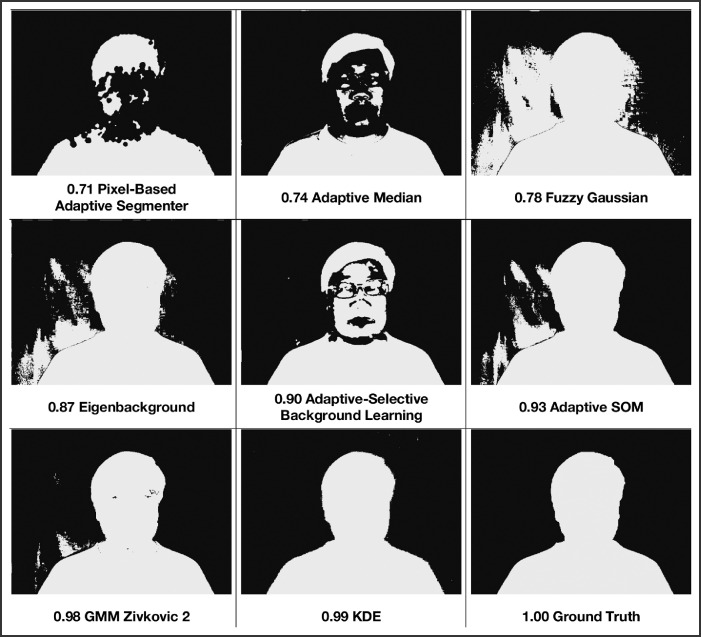
Comparison of segmentation results with the 'ground truth' for the clip taken under standard conditions at the 15 s mark, showing median F1 scores (Table 3).

Fig. 2 shows a representative frame at the mid-point of the analysis period from videos after segmentation with some of the BGSLibrary algorithms under standard conditions, with median F1 scores shown. This is so that visual inspection can pick up large areas of over and under-segmentation, and whether these areas are well demarcated from the main head and body segment. From these we can see that only the GMM Zivkovic and KDE algorithms have well-defined outlines with only small areas that have been misclassified, thus establishing the cutoff for good segmentation at an F1 score of 0.95 and higher. Similarly, the Eigenbackground, Adaptive-Selective Background Learning, and Adaptive SOM routines gave recognizable outlines for the head and upper body, with misclassified areas that are clearly separated from the main region. Hence, this establishes the cutoff for adequate segmentation at an F1 score of 0.80–0.95.

Table 1 shows a classification scheme for segmentation methods based on the approach used, which was derived from a new systematic literature review [4]. Table 2 lists the factors which affect segmentation efficacy that were obtained from the same review. The folders and files in the data repository were named using the labels in the second table. While there is a rich literature source for segmentation methods, and some of these articles provide good in-class comparisons with related routines, there is a need for an updated and comprehensive cross-class review for this topic, the last of which was published more than five years ago [3, [6], [7], [8], [9]].

**Table 1:** Classification of foreground-background segmentation methods for the head and upper body.

Non-Temporal			
	Histogram Thresholding		
	Cluster-Based		
	Region-Based		
	Deep Learning		
	Graph-Cut		
	Active Contours		
	Depth Maps		
Temporal			
	Complex recursive		
		Single Gaussian Distributions	Gaussian Average Simple Gaussian
		Gaussian Mixture Models	GMM Stauffer GMM KaewTraKulPong GMM Zivkovic 1 GMM Zivkovic 2 GMM Bender
		Non-Parametric	GMG VuMeter KDE IMBS KNN Background Subtractor SuBSENSE Pixel-Based Adaptive Segmenter ViBe Codebook PAWCS
		Histogram Density Estimates	Texture BGS Multi-Layer BGS
		Spatial Correlates	Texture-Based Foreground Detection with MRF MultiCue BGS LOBSTER Eigenbackground SL-PCA
		Fuzzy Integration	Fuzzy Sugeno Integral Fuzzy Choquet Integral Fuzzy Gaussian Type-2 Fuzzy GMM-UM Type-2 Fuzzy GMM-UV Type-2 Fuzzy GMM-UM with MRF Type-2 Fuzzy GMM-UV with MRF
		Self-Organizing Neural	Adaptive SOM Fuzzy Adaptive SOM
	Simple recursive		Weighted Moving Mean Weighted Moving Variance Adaptive Background Learning Adaptive-Selective Background Learning Temporal Mean Adaptive Median Temporal Median Sigma Delta
	Non-recursive		Static Frame Difference Frame Difference

Note: The list of segmentation methods is taken from the *C*++ wrapper ‘BGSLibrary’ v3.0.0 by Andrews Sobral [2, 3]. Some methods fall into more than one class or utilize a hybrid approach. In these cases, the class listed best describes the novelty of the approach. This library contains only temporal segmentation methods since it is designed to process video sequences.

**Table 2:** Simulated factors and modifications to the capture and processing conditions.

Factor	Clips	Labels	Capture and Processing Conditions
None	1	STD	• Standard capture conditions
Gaussian Noise	4	GAU10 GAU20 GAU30 GAU40	• Gaussian noise added independently to each color channel with a mean of zero, standard deviation of 10, 20, 30, 40, and pixel values clipped to between 0 and 255
Uniform Noise	4	UNI05 UNI10 UNI15 UNI20	• Uniform noise added independently to each color channel, distributed uniformly between 0 and 255, and replacing the value with a probability of 5%, 10%, 15%, and 20%
Camera Jitter	1	JIT	• Jitter simulated by periodically vibrating the supporting tripod with a soft mallet
Illumination	2	GLO LOC	• Global illumination changes by brightening lights • Local illumination changes by turning half the lights on one side off
Shadows	1	SHA	• Half the lights on one side were turned off throughout the clip
Background Initialization	2	INI00 INI02	• Zero initialization was simulated by trimming the clip to show the subject at the start • Short initialization was simulated by trimming the first portion of the clip to show 2 s of clear background
Color Camouflage	2	CAMSM CAMCX	• Cream-colored screen used for simple camouflage • No screen used, and background shows a house interior in daylight
Ghost Images	1	GHO	• Patterned cloth is layered over part of the background at the start, and dropped at the 10 s mark • No subject is present, and the depth data is ignored • Comparison period begins one second after the patterned cloth completely leaves the image
Sleeping Foreground	1	SLP	• Patterned cloth is lowered to cover part of the background at the 10 s mark • No subject is present, and the depth data is ignored • Comparison period begins 10 s after the patterned cloth is lowered
Dynamic Background	1	DYN	• A stand fan is placed in front of the background and turned on • No subject is present, and the depth data is ignored • Comparison period begins 10 s after the clip starts

* Where the depth data is ignored, the comparison period starts one second after the transition for the factor or 10 s after the clip starts where no transition occurs.

Note: These factors were chosen based on the literature review.

Table 3 and Table 4 give the summary results for efficacy and processing time respectively for each of the algorithms under the conditions listed in Table 2. The efficacy results are calculated frame-by-frame and the results listed consist of the median value with a 10–90th percentile range. Processing time is given as the average for all frames during the assessment period.

**Table 3:** Efficacy of segmentation algorithms from the ‘BGSLibrary’ under specific factors as F1 scores or false positive rates.

	F1 Score																	False Positive Rate		
		
		
Each cell gives efficacy as:10th CentileMedian90th Centile	STD	GAU10	GAU20	GAU30	GAU40	UNI05	UNI10	UNI15	UNI20	JIT	GLO	LOC	SHA	INI00	INI02	CAMSM	CAMCX	GHO	SLP	DYN
**Frame Difference**	0.000 0.002 0.027	0.593 0.603 0.634	0.593 0.603 0.634	0.592 0.603 0.634	0.593 0.603 0.633	0.113 0.125 0.175	0.119 0.128 0.168	0.119 0.128 0.169	0.114 0.125 0.175	0.000 0.008 0.105	0.000 0.000 0.018	0.000 0.000 0.113	0.000 0.000 0.118	0.000 0.001 0.003	0.000 0.001 0.004	0.000 0.002 0.214	0.000 0.001 0.127	0.001 0.002 0.002	0.000 0.000 0.004	0.000 0.000 0.001
**Static Frame Difference**	0.862 0.881 0.900	0.832 0.848 0.863	0.838 0.852 0.871	0.833 0.849 0.865	0.839 0.850 0.867	0.792 0.808 0.822	0.795 0.809 0.823	0.796 0.810 0.825	0.797 0.812 0.826	0.822 0.861 0.884	0.477 0.801 0.809	0.476 0.932 0.944	0.758 0.800 0.809	0.254 0.285 0.371	0.869 0.879 0.892	0.601 0.664 0.674	0.473 0.485 0.488	0.452 0.453 0.455	0.424 0.432 0.433	0.002 0.008 0.012
**Weighted Moving Mean**	0.000 0.000 0.014	0.463 0.470 0.494	0.464 0.471 0.493	0.463 0.471 0.495	0.464 0.471 0.494	0.028 0.031 0.046	0.028 0.032 0.047	0.028 0.031 0.048	0.028 0.031 0.047	0.000 0.002 0.058	0.000 0.000 0.002	0.000 0.000 0.070	0.000 0.000 0.069	0.000 0.000 0.001	0.000 0.000 0.001	0.000 0.000 0.135	0.000 0.000 0.085	0.001 0.002 0.003	0.000 0.000 0.001	0.000 0.000 0.000
**Weighted Moving Variance**	0.000 0.000 0.019	0.564 0.572 0.582	0.565 0.572 0.582	0.565 0.572 0.582	0.565 0.572 0.582	0.040 0.045 0.066	0.042 0.045 0.067	0.042 0.045 0.069	0.040 0.044 0.067	0.000 0.004 0.073	0.000 0.000 0.002	0.000 0.000 0.120	0.000 0.000 0.109	0.000 0.000 0.001	0.000 0.000 0.001	0.000 0.001 0.182	0.000 0.000 0.108	0.001 0.002 0.003	0.000 0.000 0.001	0.000 0.000 0.000
**GMM Zivkovic 1**	0.000 0.003 0.042	0.518 0.548 0.620	0.518 0.548 0.621	0.518 0.548 0.620	0.518 0.547 0.621	0.041 0.048 0.092	0.042 0.048 0.090	0.042 0.048 0.085	0.042 0.048 0.092	0.001 0.005 0.090	0.000 0.001 0.039	0.000 0.002 0.152	0.000 0.002 0.119	0.000 0.002 0.006	0.000 0.002 0.006	0.001 0.004 0.204	0.000 0.003 0.115	0.000 0.000 0.001	0.000 0.000 0.009	0.000 0.000 0.001
**Adaptive Background Learning**	0.018 0.045 0.792	0.598 0.613 0.773	0.598 0.613 0.776	0.599 0.613 0.777	0.599 0.613 0.777	0.085 0.114 0.741	0.086 0.115 0.744	0.087 0.115 0.742	0.086 0.114 0.743	0.023 0.066 0.750	0.000 0.007 0.659	0.007 0.035 0.772	0.008 0.074 0.764	0.014 0.027 0.055	0.016 0.032 0.060	0.018 0.059 0.710	0.014 0.040 0.701	0.003 0.003 0.004	0.000 0.004 0.092	0.001 0.001 0.002
**Adaptive-Selective Background Learning**	0.888 0.903 0.924	0.883 0.898 0.916	0.884 0.899 0.917	0.882 0.898 0.917	0.883 0.900 0.917	0.875 0.896 0.919	0.876 0.896 0.920	0.876 0.896 0.921	0.877 0.896 0.920	0.852 0.871 0.907	0.470 0.755 0.787	0.516 0.878 0.907	0.859 0.879 0.907	0.057 0.071 0.100	0.883 0.893 0.899	0.768 0.807 0.844	0.616 0.619 0.641	0.439 0.440 0.442	0.403 0.409 0.415	0.000 0.001 0.001
**KNN Background Subtractor**	0.000 0.002 0.021	0.561 0.576 0.631	0.561 0.576 0.628	0.561 0.576 0.631	0.561 0.576 0.630	0.031 0.037 0.077	0.032 0.038 0.077	0.032 0.038 0.075	0.032 0.037 0.076	0.000 0.004 0.077	0.000 0.000 0.022	0.000 0.000 0.047	0.000 0.001 0.072	0.000 0.001 0.003	0.000 0.001 0.003	0.000 0.001 0.128	0.000 0.001 0.093	0.000 0.001 0.001	0.000 0.000 0.004	0.000 0.000 0.001
**Adaptive Median**	0.593 0.743 0.835	0.464 0.634 0.773	0.464 0.632 0.777	0.463 0.632 0.777	0.464 0.635 0.776	0.566 0.730 0.825	0.568 0.731 0.826	0.569 0.729 0.825	0.571 0.731 0.826	0.589 0.750 0.818	0.000 0.000 0.532	0.109 0.686 0.757	0.001 0.501 0.677	0.003 0.004 0.009	0.010 0.615 0.724	0.045 0.609 0.687	0.559 0.681 0.746	0.001 0.132 0.386	0.000 0.145 0.340	0.000 0.000 0.000
**GMM Stauffer**	0.012 0.039 0.557	0.605 0.641 0.815	0.606 0.642 0.813	0.606 0.640 0.819	0.606 0.642 0.818	0.034 0.059 0.749	0.034 0.059 0.753	0.034 0.060 0.746	0.035 0.060 0.754	0.026 0.069 0.673	0.001 0.011 0.739	0.009 0.044 0.715	0.010 0.077 0.714	0.006 0.010 0.029	0.007 0.015 0.032	0.013 0.049 0.762	0.007 0.033 0.483	0.000 0.001 0.003	0.001 0.005 0.148	0.000 0.000 0.000
**GMM Zivkovic 2**	0.015 0.979 0.986	0.574 0.964 0.967	0.574 0.966 0.969	0.574 0.965 0.967	0.574 0.965 0.968	0.042 0.951 0.955	0.042 0.951 0.955	0.041 0.953 0.956	0.041 0.950 0.954	0.045 0.965 0.980	0.015 0.742 0.762	0.129 0.983 0.985	0.019 0.862 0.883	0.008 0.018 0.082	0.010 0.026 0.987	0.033 0.825 0.856	0.011 0.465 0.490	0.001 0.428 0.436	0.001 0.416 0.422	0.000 0.000 0.000
**Temporal Mean**	0.000 0.000 0.005	0.454 0.465 0.487	0.454 0.465 0.488	0.454 0.465 0.488	0.454 0.465 0.487	0.019 0.021 0.033	0.019 0.022 0.031	0.019 0.022 0.031	0.019 0.021 0.033	0.000 0.000 0.027	0.000 0.000 0.000	0.000 0.000 0.017	0.000 0.000 0.018	0.000 0.000 0.000	0.000 0.000 0.000	0.000 0.000 0.056	0.000 0.000 0.036	0.001 0.002 0.002	0.000 0.000 0.000	0.000 0.000 0.000
**Gaussian Average**	0.008 0.661 0.884	0.492 0.609 0.877	0.492 0.613 0.877	0.493 0.611 0.878	0.493 0.611 0.877	0.018 0.644 0.881	0.018 0.640 0.880	0.018 0.646 0.882	0.018 0.644 0.881	0.023 0.713 0.869	0.000 0.109 0.644	0.010 0.512 0.812	0.009 0.547 0.802	0.007 0.012 0.031	0.007 0.026 0.516	0.031 0.645 0.784	0.010 0.667 0.793	0.001 0.002 0.405	0.000 0.038 0.383	0.000 0.000 0.000
**Temporal Median**	0.006 0.018 0.937	0.550 0.560 0.940	0.550 0.560 0.941	0.550 0.560 0.940	0.550 0.560 0.941	0.015 0.026 0.937	0.015 0.027 0.939	0.015 0.026 0.939	0.015 0.026 0.935	0.008 0.031 0.909	0.000 0.001 0.463	0.001 0.011 0.857	0.001 0.024 0.792	0.003 0.010 0.022	0.005 0.011 0.027	0.008 0.034 0.785	0.003 0.024 0.795	0.000 0.000 0.000	0.000 0.001 0.274	0.000 0.000 0.000
**Eigenbackground SL-PCA**	0.854 0.872 0.892	0.835 0.852 0.870	0.838 0.854 0.880	0.838 0.856 0.873	0.839 0.854 0.874	0.820 0.843 0.860	0.828 0.844 0.861	0.829 0.845 0.862	0.824 0.842 0.859	0.797 0.835 0.858	0.486 0.887 0.892	0.512 0.956 0.965	0.802 0.837 0.843	0.211 0.240 0.324	0.849 0.858 0.871	0.680 0.784 0.802	0.487 0.498 0.501	0.455 0.457 0.458	0.423 0.433 0.435	0.000 0.000 0.002
**Texture BGS**	0.529 0.551 0.604	0.178 0.195 0.239	0.172 0.192 0.240	0.172 0.193 0.237	0.168 0.188 0.242	0.238 0.284 0.388	0.234 0.283 0.384	0.257 0.301 0.391	0.261 0.307 0.396	0.530 0.548 0.614	0.197 0.237 0.538	0.491 0.520 0.612	0.572 0.593 0.663	0.017 0.034 0.093	0.529 0.538 0.548	0.651 0.662 0.683	0.646 0.669 0.715	0.423 0.424 0.426	0.407 0.411 0.414	0.000 0.000 0.000
**Type-2 Fuzzy GMM-UM**	0.000 0.000 0.655	0.000 0.000 0.324	0.000 0.000 0.324	0.000 0.000 0.331	0.000 0.000 0.328	0.000 0.000 0.219	0.000 0.000 0.216	0.000 0.000 0.226	0.000 0.000 0.234	0.000 0.000 0.310	0.000 0.000 0.010	0.000 0.000 0.535	0.000 0.000 0.386	0.000 0.000 0.000	0.000 0.000 0.000	0.000 0.000 0.007	0.000 0.000 0.262	0.000 0.000 0.000	0.000 0.000 0.010	0.000 0.000 0.000
**Type-2 Fuzzy GMM-UV**	0.054 0.118 0.737	0.696 0.718 0.857	0.693 0.720 0.858	0.696 0.721 0.859	0.694 0.718 0.859	0.370 0.417 0.812	0.377 0.423 0.813	0.374 0.421 0.810	0.369 0.412 0.809	0.101 0.175 0.821	0.028 0.104 0.639	0.048 0.138 0.855	0.066 0.204 0.818	0.031 0.044 0.072	0.040 0.071 0.109	0.060 0.127 0.729	0.036 0.097 0.825	0.001 0.004 0.019	0.015 0.038 0.278	0.004 0.005 0.006
**Type-2 Fuzzy GMM-UM with MRF**	0.000 0.000 0.395	0.000 0.000 0.139	0.000 0.000 0.137	0.000 0.000 0.137	0.000 0.000 0.142	0.000 0.000 0.104	0.000 0.000 0.103	0.000 0.000 0.109	0.000 0.000 0.115	0.000 0.000 0.192	0.000 0.000 0.000	0.000 0.000 0.299	0.000 0.000 0.146	0.000 0.000 0.000	0.000 0.000 0.000	0.000 0.000 0.002	0.000 0.000 0.202	0.000 0.000 0.000	0.000 0.000 0.008	0.000 0.000 0.000
**Type-2 Fuzzy GMM-UV with MRF**	0.011 0.037 0.677	0.522 0.536 0.694	0.524 0.536 0.695	0.523 0.537 0.696	0.524 0.537 0.692	0.035 0.070 0.639	0.033 0.069 0.640	0.034 0.071 0.648	0.034 0.073 0.646	0.025 0.062 0.728	0.002 0.009 0.438	0.008 0.044 0.803	0.012 0.074 0.698	0.006 0.011 0.022	0.007 0.015 0.029	0.009 0.031 0.593	0.006 0.018 0.635	0.000 0.000 0.003	0.002 0.009 0.150	0.000 0.001 0.001
**Fuzzy Sugeno Integral**	0.100 0.164 0.634	0.096 0.183 0.395	0.099 0.183 0.394	0.097 0.183 0.394	0.098 0.182 0.398	0.094 0.165 0.655	0.093 0.166 0.656	0.094 0.166 0.655	0.095 0.166 0.653	0.102 0.152 0.652	0.150 0.194 0.698	0.078 0.143 0.237	0.052 0.141 0.588	0.006 0.011 0.027	0.024 0.126 0.156	0.147 0.184 0.668	0.041 0.306 0.644	0.000 0.006 0.248	0.008 0.051 0.252	0.000 0.000 0.000
**Fuzzy Choquet Integral**	0.125 0.171 0.603	0.173 0.269 0.499	0.174 0.267 0.495	0.174 0.268 0.499	0.173 0.268 0.499	0.128 0.176 0.665	0.127 0.177 0.669	0.127 0.177 0.668	0.127 0.177 0.666	0.119 0.156 0.646	0.174 0.309 0.706	0.099 0.139 0.408	0.064 0.151 0.574	0.008 0.013 0.030	0.054 0.139 0.163	0.160 0.198 0.660	0.050 0.441 0.604	0.001 0.011 0.283	0.012 0.077 0.296	0.000 0.000 0.000
**Simple Gaussian**	0.719 0.768 0.810	0.708 0.751 0.787	0.707 0.760 0.791	0.708 0.754 0.789	0.714 0.755 0.791	0.686 0.723 0.746	0.687 0.726 0.747	0.691 0.729 0.751	0.692 0.729 0.751	0.693 0.762 0.808	0.486 0.916 0.963	0.486 0.820 0.853	0.690 0.749 0.781	0.307 0.351 0.424	0.779 0.799 0.836	0.611 0.699 0.747	0.482 0.495 0.498	0.462 0.470 0.481	0.433 0.441 0.462	0.005 0.013 0.019
**Fuzzy Gaussian**	0.738 0.782 0.807	0.720 0.761 0.783	0.721 0.768 0.787	0.720 0.762 0.784	0.725 0.765 0.787	0.700 0.734 0.754	0.699 0.736 0.755	0.703 0.739 0.757	0.703 0.737 0.757	0.713 0.774 0.802	0.495 0.916 0.941	0.465 0.833 0.856	0.707 0.767 0.788	0.287 0.320 0.372	0.786 0.800 0.816	0.605 0.669 0.704	0.483 0.495 0.497	0.461 0.464 0.472	0.431 0.438 0.450	0.004 0.011 0.016
**GMM Bender**	0.034 0.071 0.927	0.625 0.672 0.944	0.626 0.673 0.945	0.626 0.673 0.944	0.625 0.673 0.946	0.101 0.156 0.892	0.101 0.156 0.894	0.101 0.158 0.894	0.101 0.155 0.889	0.048 0.102 0.872	0.003 0.027 0.795	0.019 0.078 0.954	0.030 0.129 0.873	0.024 0.044 0.082	0.026 0.048 0.079	0.035 0.116 0.770	0.019 0.057 0.493	0.000 0.001 0.003	0.002 0.015 0.411	0.001 0.001 0.002
**Adaptive SOM**	0.859 0.927 0.935	0.910 0.961 0.968	0.910 0.961 0.967	0.908 0.959 0.966	0.910 0.960 0.966	0.878 0.949 0.964	0.879 0.951 0.965	0.877 0.950 0.965	0.875 0.950 0.964	0.808 0.907 0.930	0.485 0.807 0.875	0.600 0.971 0.976	0.818 0.884 0.894	0.108 0.129 0.169	0.947 0.950 0.952	0.824 0.943 0.948	0.484 0.492 0.494	0.452 0.453 0.455	0.424 0.428 0.429	0.000 0.000 0.000
**Fuzzy Adaptive SOM**	0.897 0.919 0.934	0.943 0.954 0.964	0.942 0.955 0.965	0.940 0.953 0.963	0.944 0.956 0.964	0.881 0.909 0.923	0.888 0.906 0.919	0.889 0.906 0.919	0.880 0.905 0.918	0.830 0.868 0.903	0.480 0.807 0.821	0.528 0.959 0.968	0.808 0.846 0.852	0.130 0.164 0.240	0.988 0.989 0.990	0.798 0.916 0.928	0.479 0.490 0.493	0.452 0.453 0.454	0.422 0.432 0.433	0.000 0.000 0.000
**Texture-Based Foreground Detection with MRF**	0.024 0.111 0.499	0.714 0.744 0.902	0.715 0.743 0.894	0.716 0.744 0.910	0.715 0.745 0.921	0.535 0.615 0.687	0.537 0.603 0.698	0.534 0.611 0.693	0.524 0.608 0.681	0.094 0.192 0.608	0.026 0.068 0.401	0.022 0.118 0.685	0.064 0.243 0.710	0.002 0.015 0.057	0.012 0.042 0.108	0.046 0.145 0.694	0.005 0.078 0.745	0.015 0.036 0.121	0.001 0.007 0.396	0.000 0.001 0.001
**Multi-Layer BGS**	0.000 0.001 0.172	0.239 0.302 0.726	0.237 0.298 0.726	0.240 0.300 0.726	0.237 0.303 0.725	0.033 0.062 0.266	0.029 0.061 0.264	0.031 0.060 0.264	0.031 0.061 0.266	0.000 0.003 0.234	0.000 0.000 0.267	0.000 0.000 0.671	0.000 0.000 0.513	0.000 0.000 0.007	0.000 0.000 0.007	0.000 0.001 0.550	0.000 0.000 0.410	0.000 0.000 0.001	0.000 0.000 0.013	0.000 0.000 0.000
**Pixel-Based Adaptive Segmenter**	0.539 0.714 0.978	0.398 0.577 0.944	0.406 0.582 0.948	0.418 0.596 0.947	0.410 0.584 0.952	0.266 0.440 0.790	0.262 0.443 0.789	0.247 0.434 0.788	0.252 0.432 0.794	0.400 0.678 0.966	0.326 0.435 0.759	0.238 0.543 0.977	0.000 0.119 0.914	0.000 0.000 0.000	0.456 0.582 0.685	0.084 0.337 0.914	0.403 0.654 0.879	0.174 0.275 0.405	0.313 0.366 0.409	0.000 0.000 0.000
**VuMeter**	0.000 0.000 0.080	0.000 0.000 0.185	0.000 0.000 0.199	0.000 0.000 0.193	0.000 0.000 0.193	0.000 0.000 0.071	0.000 0.000 0.069	0.000 0.000 0.071	0.000 0.000 0.074	0.000 0.000 0.217	0.000 0.000 0.304	0.000 0.000 0.558	0.000 0.000 0.522	0.000 0.000 0.000	0.000 0.000 0.000	0.000 0.000 0.517	0.000 0.000 0.370	0.000 0.000 0.000	0.000 0.000 0.005	0.000 0.000 0.000
**KDE**	0.492 0.993 0.994	0.650 0.960 0.972	0.661 0.956 0.974	0.657 0.961 0.974	0.655 0.959 0.973	0.505 0.971 0.979	0.507 0.972 0.980	0.505 0.972 0.980	0.504 0.971 0.979	0.406 0.992 0.994	0.079 0.932 0.946	0.881 0.990 0.994	0.821 0.964 0.969	0.110 0.121 0.132	0.111 0.993 0.995	0.653 0.959 0.982	0.594 0.854 0.858	0.002 0.435 0.439	0.092 0.413 0.414	0.002 0.003 0.004
**IMBS**	0.002 0.481 0.997	0.625 0.812 0.993	0.625 0.812 0.993	0.624 0.814 0.993	0.624 0.818 0.993	0.004 0.908 0.996	0.004 0.912 0.996	0.004 0.911 0.996	0.004 0.912 0.996	0.028 0.281 0.997	0.003 0.103 0.984	0.008 0.945 0.995	0.003 0.831 0.959	0.000 0.008 0.029	0.000 0.017 0.049	0.052 0.453 0.976	0.001 0.745 0.803	0.000 0.000 0.453	0.001 0.383 0.412	0.000 0.000 0.000
**MultiCue BGS**	0.011 0.044 0.929	0.760 0.915 0.931	0.766 0.919 0.931	0.762 0.916 0.931	0.765 0.905 0.932	0.411 0.887 0.922	0.381 0.886 0.926	0.402 0.883 0.924	0.431 0.890 0.925	0.183 0.320 0.928	0.000 0.238 0.302	0.000 0.874 0.916	0.214 0.859 0.912	0.000 0.000 0.031	0.213 0.287 0.453	0.360 0.883 0.911	0.200 0.617 0.651	0.000 0.000 0.000	0.120 0.352 0.420	0.000 0.000 0.000
**Sigma Delta**	0.016 0.057 0.946	0.571 0.592 0.929	0.570 0.591 0.931	0.571 0.591 0.927	0.572 0.592 0.929	0.120 0.168 0.881	0.121 0.171 0.878	0.121 0.171 0.882	0.122 0.169 0.877	0.023 0.082 0.958	0.000 0.007 0.743	0.007 0.065 0.970	0.006 0.067 0.900	0.011 0.025 0.055	0.013 0.030 0.061	0.017 0.062 0.928	0.014 0.045 0.851	0.000 0.002 0.093	0.000 0.005 0.276	0.001 0.001 0.002
**SuBSENSE**	0.002 0.004 0.396	0.531 0.546 0.718	0.560 0.568 0.711	0.529 0.542 0.705	0.534 0.544 0.726	0.000 0.000 0.005	0.000 0.000 0.006	0.000 0.000 0.007	0.000 0.000 0.003	0.016 0.020 0.453	0.000 0.734 0.914	0.000 0.000 0.963	0.543 0.709 0.969	0.000 0.000 0.000	0.004 0.005 0.005	0.014 0.017 0.898	0.000 0.000 0.194	0.000 0.000 0.000	0.000 0.000 0.142	0.000 0.000 0.000
**LOBSTER**	0.940 0.973 0.986	0.921 0.964 0.985	0.924 0.963 0.985	0.921 0.962 0.984	0.921 0.964 0.985	0.880 0.945 0.983	0.885 0.947 0.983	0.891 0.948 0.984	0.892 0.945 0.983	0.909 0.956 0.987	0.500 0.933 0.984	0.440 0.836 0.918	0.683 0.774 0.872	0.008 0.017 0.042	0.918 0.950 0.970	0.646 0.706 0.800	0.666 0.739 0.789	0.368 0.400 0.420	0.375 0.399 0.406	0.000 0.000 0.000
**PAWCS**	0.000 0.111 0.979	0.539 0.606 0.980	0.533 0.625 0.981	0.532 0.598 0.981	0.521 0.593 0.980	0.000 0.000 0.089	0.000 0.000 0.300	0.000 0.000 0.442	0.000 0.000 0.094	0.000 0.094 0.980	0.317 0.927 0.973	0.000 0.108 0.985	0.000 0.122 0.966	0.000 0.000 0.000	0.000 0.334 0.636	0.000 0.047 0.973	0.000 0.000 0.831	0.000 0.000 0.313	0.000 0.015 0.420	0.000 0.000 0.000
**TwoPoints**	0.545 0.700 0.860	0.346 0.530 0.796	0.338 0.512 0.791	0.343 0.526 0.795	0.341 0.511 0.793	0.441 0.635 0.821	0.462 0.645 0.821	0.420 0.627 0.818	0.418 0.632 0.818	0.554 0.703 0.853	0.407 0.532 0.720	0.348 0.652 0.819	0.026 0.276 0.797	0.011 0.017 0.032	0.469 0.597 0.678	0.306 0.527 0.787	0.415 0.629 0.725	0.001 0.012 0.235	0.005 0.134 0.365	0.001 0.001 0.001
**ViBe**	0.696 0.728 0.769	0.498 0.594 0.700	0.497 0.592 0.702	0.493 0.595 0.712	0.496 0.596 0.694	0.617 0.705 0.768	0.608 0.707 0.772	0.632 0.708 0.770	0.635 0.709 0.771	0.689 0.739 0.792	0.460 0.586 0.656	0.348 0.723 0.757	0.561 0.669 0.749	0.002 0.004 0.010	0.667 0.693 0.714	0.607 0.688 0.770	0.660 0.721 0.755	0.036 0.146 0.284	0.018 0.073 0.246	0.000 0.000 0.000
**Codebook**	0.146 0.461 0.728	0.636 0.738 0.797	0.636 0.739 0.797	0.635 0.737 0.796	0.635 0.739 0.802	0.260 0.582 0.742	0.260 0.587 0.746	0.257 0.581 0.747	0.260 0.576 0.742	0.229 0.514 0.717	0.135 0.492 0.867	0.206 0.465 0.737	0.221 0.624 0.751	0.143 0.210 0.318	0.125 0.208 0.390	0.289 0.588 0.731	0.129 0.455 0.568	0.004 0.021 0.469	0.044 0.246 0.454	0.002 0.005 0.010

Note: The segmentation algorithms are listed in Table 1 while the labels for the factors are listed in Table 2. Two routines were not formally tested (GMM KaewTraKulPong and GMG) as they required an earlier version of OpenCV. The efficacy measures are calculated on a per-frame basis, and the three numbers listed in each cell are 10th centile, median, and 90th centile.

**Table 4:** Processing times of segmentation algorithms from the ‘BGSLibrary’ in milliseconds per frame.

	Processing Time Per Frame (ms/frame)																			
	STD	GAU10	GAU20	GAU30	GAU40	UNI05	UNI10	UNI15	UNI20	JIT	GLO	LOC	SHA	INI00	INI02	CAMSM	CAMCX	GHO	SLP	DYN
**Frame Difference**	2.3	1.4	2.3	3.4	5.3	7.5	10.1	14.6	2.2	1.9	2.0	1.6	4.4	1.8	1.9	1.9	2.6	5.0	5.9	6.6
**Static Frame Difference**	2.2	1.4	2.2	3.2	5.2	7.4	10.0	14.1	2.3	1.8	2.0	1.8	4.2	1.7	1.8	1.8	2.4	4.9	5.8	6.5
**Weighted Moving Mean**	20.6	16.9	18.8	20.7	22.7	25.0	29.9	39.5	21.6	16.7	18.7	16.7	20.3	17.9	17.8	17.2	18.4	21.3	22.9	24.2
**Weighted Moving Variance**	56.5	55.0	51.6	56.4	59.1	70.1	77.7	96.5	55.6	50.5	54.3	50.1	55.8	53.1	53.1	52.3	54.0	57.7	64.7	61.7
**GMM Zivkovic 1**	12.7	15.2	14.6	16.4	15.9	16.0	16.9	18.7	13.0	12.7	12.5	11.6	13.4	12.4	11.9	12.8	13.9	13.6	14.6	14.4
**Adaptive Background Learning**	14.1	14.9	13.8	16.4	18.3	27.5	30.4	37.0	13.4	15.0	13.7	14.1	18.1	15.2	14.9	15.8	17.3	19.4	21.8	22.1
**Adaptive-Selective Background Learning**	6.4	5.3	6.5	8.6	10.5	15.5	18.6	24.7	6.0	5.9	6.1	5.1	8.7	5.5	5.6	6.1	6.3	9.4	11.9	11.4
**KNN Background Subtractor**	50.2	61.4	53.8	53.6	54.5	54.9	56.1	77.0	56.3	47.7	51.6	50.0	48.7	49.3	52.0	52.1	52.1	49.6	51.4	49.5
**Adaptive Median**	4.0	4.6	4.2	4.5	4.9	5.8	6.9	9.2	4.3	4.1	4.1	4.0	4.5	4.1	4.3	4.2	4.4	4.8	5.1	5.2
**GMM Stauffer**	43.8	62.7	56.2	53.5	56.1	56.0	57.6	78.9	62.0	44.7	43.3	46.1	45.4	31.7	46.4	47.6	49.3	46.7	47.1	32.6
**GMM Zivkovic 2**	12.6	15.1	14.1	13.3	14.4	15.7	16.0	23.5	16.0	13.0	14.5	14.2	13.8	12.2	13.1	14.7	15.9	14.1	13.5	13.6
**Temporal Mean**	9.1	10.1	9.6	9.3	10.0	10.5	11.3	13.7	9.9	10.0	9.6	9.3	10.6	9.3	9.3	10.7	10.7	10.5	10.7	10.6
**Gaussian Average**	8.4	9.3	8.9	8.7	9.6	10.1	10.8	12.8	9.2	9.2	8.6	8.6	9.8	8.8	8.5	9.8	9.7	9.5	9.8	10.1
**Temporal Median**	39.4	39.3	37.5	38.2	37.3	39.2	39.3	42.2	41.9	37.1	37.9	38.0	36.8	37.6	38.0	43.9	39.3	36.7	38.5	38.2
**Eigenbackground SL-PCA**	44.6	44.6	41.0	42.9	43.2	47.3	49.7	54.2	47.1	43.4	43.2	43.5	44.6	43.9	42.9	49.4	45.9	44.1	47.1	47.4
**Texture BGS**	334.1	314.2	314.3	305.2	305.0	321.7	311.2	338.6	348.1	325.6	330.1	328.9	319.3	329.9	337.6	343.0	345.7	303.4	309.3	335.9
**Type-2 Fuzzy GMM-UM**	47.1	45.3	44.8	45.6	44.1	51.3	51.6	56.5	59.4	45.4	42.6	46.0	42.7	37.0	46.3	49.3	47.8	45.5	47.5	42.3
**Type-2 Fuzzy GMM-UV**	55.0	62.9	62.4	60.9	62.0	65.0	64.6	70.3	68.7	55.4	54.7	54.8	54.0	53.3	59.0	61.9	59.9	56.7	57.1	55.1
**Type-2 Fuzzy GMM-UM with MRF**	84.6	82.1	76.9	78.1	77.0	85.7	85.0	92.2	90.3	79.9	74.4	78.1	76.3	71.7	82.0	84.8	82.9	79.3	82.1	70.8
**Type-2 Fuzzy GMM-UV with MRF**	92.1	97.4	96.2	91.9	92.0	98.4	97.8	103.0	102.1	86.4	84.0	87.2	85.1	80.7	89.8	92.9	88.4	88.6	87.9	81.3
**Fuzzy Sugeno Integral**	183.1	163.8	177.3	171.1	166.3	175.6	176.5	186.8	196.0	177.8	173.8	175.4	172.1	167.0	182.9	182.0	187.7	173.1	175.6	170.6
**Fuzzy Choquet Integral**	178.9	172.0	174.4	167.3	169.6	180.7	176.8	195.4	190.3	177.1	170.7	171.7	171.6	175.2	186.0	178.9	187.3	175.3	174.9	173.0
**Simple Gaussian**	9.0	9.8	11.3	10.7	11.9	13.0	14.3	16.4	11.3	10.2	8.7	9.8	10.7	9.3	10.1	10.8	10.4	10.7	10.8	12.0
**Fuzzy Gaussian**	18.8	19.1	19.0	19.8	20.1	21.5	22.9	25.1	20.1	19.3	17.2	17.6	19.6	20.2	18.2	18.6	18.5	19.9	20.2	22.9
**GMM Bender**	21.7	22.6	21.9	22.4	23.2	27.6	26.6	31.0	25.1	23.3	22.3	22.5	24.3	22.7	24.9	24.0	25.3	23.5	23.5	24.9
**Adaptive SOM**	40.7	41.1	41.8	40.9	43.3	45.2	46.0	53.1	45.8	40.7	33.3	36.8	44.6	52.9	43.0	45.2	36.1	44.2	43.8	53.0
**Fuzzy Adaptive SOM**	53.1	49.9	51.9	53.1	53.1	54.3	56.5	63.0	59.5	52.5	55.0	50.8	56.3	56.6	53.4	55.8	59.3	56.7	57.4	59.0
**Texture-Based Foreground Detection with MRF**	242.1	237.8	233.0	248.0	274.4	328.2	359.5	404.7	295.3	250.5	239.6	244.3	300.2	244.0	251.9	265.0	255.1	304.2	303.8	307.3
**Multi-Layer BGS**	208.1	249.4	249.2	251.9	265.2	285.5	302.6	328.2	276.3	207.2	214.4	208.1	228.6	187.3	220.2	235.1	210.2	230.3	235.9	211.1
**Pixel-Based Adaptive Segmenter**	124.3	114.0	112.3	112.7	121.0	112.1	119.8	134.9	118.0	118.2	160.5	115.2	105.0	86.5	140.1	115.5	125.5	147.3	158.3	93.8
**VuMeter**	13.3	14.1	14.9	15.1	16.8	19.1	21.7	24.5	15.7	14.2	13.8	14.4	18.7	14.0	14.0	15.2	15.4	18.6	20.5	19.6
**KDE**	31.0	39.9	41.5	41.9	47.0	43.4	42.8	47.4	48.2	28.6	54.7	34.9	31.3	18.8	31.5	32.1	38.3	36.4	33.1	18.3
**IMBS**	42.9	54.6	58.6	65.5	81.3	94.2	112.3	129.7	67.6	42.9	42.2	54.2	50.9	29.5	33.4	52.3	49.5	50.6	46.9	35.5
**MultiCue BGS**	36.9	37.2	40.5	43.0	53.4	64.5	71.0	83.9	47.5	34.7	34.6	33.3	44.3	30.3	40.0	40.1	35.9	38.4	48.6	39.6
**Sigma Delta**	11.2	10.9	11.2	12.2	14.8	17.8	18.1	21.0	12.4	11.0	10.4	10.6	11.8	11.1	12.0	12.2	10.3	12.7	13.2	15.4
**SuBSENSE**	346.5	376.0	417.3	423.8	416.9	365.9	379.7	379.7	453.3	297.7	400.7	317.8	328.2	288.3	313.2	325.0	299.4	287.6	311.1	279.3
**LOBSTER**	256.0	240.4	282.0	282.7	280.6	228.0	230.4	264.2	279.5	223.5	234.3	192.4	203.0	212.5	244.4	197.2	204.9	197.0	189.9	221.2
**PAWCS**	542.8	855.7	651.6	650.3	649.5	829.9	829.1	904.0	692.2	711.5	667.1	753.3	703.6	597.5	763.5	789.0	840.6	722.4	758.2	567.3
**TwoPoints**	4.0	4.1	5.0	5.9	7.6	8.8	11.2	14.9	4.6	3.9	3.7	3.6	6.0	3.8	3.9	3.9	4.6	6.6	7.9	8.4
**ViBe**	8.7	9.5	7.8	8.3	9.2	11.4	12.1	14.5	9.0	10.4	15.5	11.7	9.5	5.6	12.1	9.7	11.0	10.1	8.9	6.3
**Codebook**	20.2	48.7	34.6	35.2	37.0	50.6	51.1	52.5	40.8	27.7	24.1	33.1	29.1	20.6	30.9	41.0	32.8	21.3	26.2	16.1

Note: The segmentation algorithms are listed in Table 1 while the labels for the factors are listed in Table 2. Two routines were not formally tested (GMM KaewTraKulPong and GMG) as they required an earlier version of OpenCV. The processing time is calculated as an average of all frames during the comparison period for that clip.

Table 5 gives a qualitative assessment of each routine's performance according to a set of criteria. This table allows routines to be chosen based on the requirements for a particular use case. It is expected that most readers would utilize this table when evaluating the merits of each routine, with the previous two tables included for reference if further details are needed.

**Table 5:** An assessment of segmentation algorithms from the ‘BGSLibrary’ according to pre-set criteria.

	High Efficacy	Consistency	Processing Speed (30 fps)	Processing Speed (10 fps)	Noise	Jitter	Global Illumination	Local Illumination	Shadows	No Initialization	short initialization	color camouflage	Complex Background	Ghost Images	Sleeping Background	Dynamic Background
**Frame Difference**			**X**	**X**										**X**	**I**	**X**
**Static Frame Difference**		**X**	**X**	**X**	**X**	**X**	**X**	**X**	**X**		**X**					
**Weighted Moving Mean**			**X**	**X**										**X**	**I**	**X**
**Weighted Moving Variance**				**X**										**X**	**I**	**X**
**GMM Zivkovic 1**			**X**	**X**										**X**	**I**	**X**
**Adaptive Background Learning**			**X**	**X**										**X**	**I**	**X**
**Adaptive-Selective Background Learning**		**X**	**X**	**X**	**X**	**X**		**X**	**X**		**X**	**X**				**X**
**KNN Background Subtractor**				**X**										**X**	**I**	**X**
**Adaptive Median**			**X**	**X**											**I**	**X**
**GMM Stauffer**				**X**										**X**	**I**	**X**
**GMM Zivkovic 2**	**X**		**X**	**X**	**X**	**X**		**X**	**X**			**X**			**I**	**X**
**Temporal Mean**			**X**	**X**										**X**	**I**	**X**
**Gaussian Average**			**X**	**X**											**I**	**X**
**Temporal Median**				**X**										**X**	**I**	**X**
**Eigenbackground SL-PCA**		**X**		**X**	**X**	X	**X**	**X**	**X**		**X**					**X**
**Texture BGS**																**X**
**Type-2 Fuzzy GMM-UM**				**X**										**X**	**I**	**X**
**Type-2 Fuzzy GMM-UV**				**X**										**X**	**I**	
**Type-2 Fuzzy GMM-UM with MRF**				**X**										**X**	**I**	**X**
**Type-2 Fuzzy GMM-UV with MRF**				**X**										**X**	**I**	**X**
**Fuzzy Sugeno Integral**															**I**	**X**
**Fuzzy Choquet Integral**															**I**	**X**
**Simple Gaussian**			**X**	**X**			**X**	**X**								
**Fuzzy Gaussian**			**X**	**X**			**X**	**X**			**X**					
**GMM Bender**			**X**	**X**										**X**	**I**	**X**
**Adaptive SOM**		**X**		**X**	**X**	**X**	**X**	**X**	**X**		**X**	**X**				**X**
**Fuzzy Adaptive SOM**		**X**		**X**	**X**	**X**	**X**	**X**	**X**		**X**	**X**				**X**
**Texture-Based Foreground Detection with MRF**															**I**	**X**
**Multi-Layer BGS**														**X**	**I**	**X**
**Pixel-Based Adaptive Segmenter**																**X**
**VuMeter**			**X**	**X**										**X**	**I**	**X**
**KDE**	**X**		**X**	**X**	**X**	**X**	**X**	**X**	**X**		**X**	**X**	**X**			
**IMBS**				**X**	**X**			**X**	**X**						**I**	**X**
**MultiCue BGS**				**X**	**X**			**X**	**X**			**X**		**X**		**X**
**Sigma Delta**			**X**	**X**											**I**	**X**
**SuBSENSE**														**X**	**I**	**X**
**LOBSTER**	**X**	**X**			**X**	**X**	**X**	**X**			**X**					**X**
**PAWCS**							**X**								**I**	**X**
**TwoPoints**			**X**	**X**											**I**	**X**
**ViBe**			**X**	**X**											**I**	**X**
**Codebook**			**X**	**X**												

Note: **X** – fulfills criteria, **I** – integrates into background.

### 1.1 Video clips

The video clips in the Mendeley Data repository are organized into folders with names according to the labels in Table 2. The individual clips are named according to the segmentation methods listed in Table 1. Tables 3 and 4 give the segmentation efficacy and processing time for each method, with one of the factors applied. Table 5 gives an assessment of the segmentation algorithms according to a set of criteria.

The ‘ground truth’ video clips were saved to AVI files encoded with the lossless FFV1 codec so that the individual frames can be extracted for analysis. These files can be viewed using VLC media player (cross-platform), Media Player Classic (Windows 10), or any player which can use the FFV1 video codec.

## 2 Experimental Design, Materials and Methods

### 2.1 Common factors affecting segmentation efficacy

There are several common factors which affect indoor segmentation efficacy: 1) image noise, 2) camera jitter and movement, 3) automatic camera settings, 4) illumination and shadows, 5) background initialization, 6) color camouflage, and 7) ghost images, sleeping foregrounds, and dynamic backgrounds [10], [11], [12].

### 2.2 Algorithm comparison

This experiment was carried out to assess the suitability of the algorithms in the *C*++ wrapper ‘BGSLibrary’ v3.0.0 developed by Andrews Sobral for head segmentation, given that it is a mature library that has been continually updated over the past eight years and now includes over 40 routines [2, 3].

The camera chosen for this features paired color-depth image sensors, with the color sensor having specifications typical of mobile devices and webcams, which cover most of the use cases for these algorithms. The factors chosen for testing are derived from the literature review as being able to influence the efficacy of background segmentation. The assessment criteria for the results were set based on the requirements of common use cases such as gamecasting and mobile communications to allow the readers to make their own judgements on the merits of each algorithm for their own purposes.

### 2.3 Methodology

A series of video clips was captured with an Intel RealSense Depth Camera D435 featuring a global shutter, large color pixels of 3 um square, and a depth sensor using disparity mapping from stereo infra-red cameras. The following camera settings were used: structured light projector on, autofocus enabled, autoexposure disabled, automatic white balancing disabled, backlight compensation disabled, and powerline frequency compensation disabled.

Capture resolution was 640 × 480 pixels at 30 frames per second (fps) for color data and 90 fps for depth data, with the depth data processed using temporal and spatial smoothing with hole-filling to reduce artefacts. Synthetic paired color and depth frames were motion interpolated from the source frames to generate video clips without any inter-frames. The clips were then saved to Audio Video Interleave (AVI) files using FFMPEG, with dimensions of 640 ×480 pixels at 30 fps. Color clips were encoded using the lossy MPEG-4 Part 2 codec at a bit rate of 4 megabit per second (Mbps) except for the noise clips which were encoded at 12 Mbps to preserve the noise artefacts.

The clips were captured at night under controlled bidirectional diagonal and side lighting with Philips Hue White & Color Ambiance bulbs set to the ‘Energize’ preset with a color temperature of 6410 K, calculated from the Mired Color Temperature supplied by the Philips Hue Software Development Kit. The camera was placed 120 cm in front of either a plain green screen (standard), a cream-colored screen (camouflage), or with the screen removed (complex), having the subject standing 60 cm in front of the screen, with no intervening objects. This resulted in a foreground area that was consistently about half the total background size, which is sufficiently balanced to not distort the measures of segmentation efficacy, and yet not too big which would prevent the face detection routine from working properly. The factors listed in Table 2 were then applied.

All clips were 40 s long with the first 10 s showing just the background. The subject entered the scene at the 10 s mark and stood in the center of the frame while keeping a neutral expression, with the face and upper body fully visible. The comparison period was set to all frames between the 20 and 40 s mark inclusive. The brightness for all clips was normalized by applying the appropriate constant gamma correction to keep the average pixel brightness throughout the clip at 50% of the maximum brightness.

For lighting change factors, the lighting conditions were altered mid-way through the comparison period. For the ‘Ghost Images’ clip, the comparison period was set to one second after the transition. This is because most algorithms for detection and removal of moving objects, ghosts, and shadows operate in less than a second. The comparison period for the ‘Sleeping Foreground’ clip was set to 10 s after the transition to allow enough time for the object to be incorporated into the background model.

In clips where a subject was present, the face location was determined using libfacedetection by Shiqi Yu, and a seed point and depth obtained from the center of the bounding rectangle [13]. The ‘ground truth’ foreground was then extracted from a floating range flood fill starting at the seed point, with a maximum difference of 2 cm between adjacent pixels. Since the depth data from disparity mapping is coarse and lacks edge accuracy, an automated GrabCut algorithm was used to refine the edges of the foreground. The ‘ground truth’ clips were verified by visually inspecting at 4 fps, and adjustments were made with manual GrabCut assistance where the areas of inaccuracy exceeded 5% of the foreground (Fig. 1). For clips without a subject, the depth data was ignored and the ‘ground truth’ foreground was set to zero.

The segmentation foreground was obtained by processing the color data using the appropriate ‘BGSLibrary’ algorithm with default settings, except for the ‘GMG’ and ‘GMM KaewTraKulPong’ routines which require an earlier version of OpenCV (see Table 1). To speed up processing, the segmentation routines were run in four isolated parallel threads in ‘release’ mode. The ‘ground truth’ and segmentation clips were saved to AVI files encoded with the lossless FFV1 codec.

Testing was performed on a system using Microsoft Windows 10 with a four-core Intel Xeon E3 processor running at 3.5 GHz using Visual Basic and Visual *C*++ 2019 in a 64-bit address space with 32 GB RAM allocated and an NVIDIA GeForce GTX 1060 6GB graphics card installed. Image processing was done using the libraries in EmguCV 3.2.0 and Accord.Net 3.8.0. The system speed was rated at 475 million floating points per second (MFLOPS) using the Intel processor diagnostic tool 2.10, 64-bit version.

Processing time was calculated as the average time taken to process each frame over the comparison period.

Accuracy was defined as follows:

(1) $$Accuracy=\frac{1}{N}\left(\sum_{t\in \left(20,\;30\right)}\frac{TP_{t}+TN_{t}}{TP_{t}+TN_{t}+FP_{t}+FN_{t}}\right)$$

In Eq. (1), *TP* (true positives) represent correctly classified foreground pixels, *TN* (true negatives) represent correctly classified background pixels, *FP* (false positives) represent incorrectly classified foreground pixels, *FN* (false negatives) represent incorrectly classified background pixels, N represents the total number of frames, and *t* represents the frame time. The ‘ground truth’ is used as the reference for both TP and TN pixels, while the comparison was the segmentation result from the ‘BGSLibrary’ algorithm. A lower result for the accuracy indicates greater deviation from the ‘ground truth’.

Precision and recall were defined as follows:

(2) $$Precision=\frac{1}{N}\left(\sum_{t\in \left(20,\;30\right)}\frac{TP_{t}}{TP_{t}+FP_{t}}\right)$$

In Eq. (2), precision refers to the proportion of the segmented foreground that has been correctly segmented.

(3) $$Recall=\frac{1}{N}\left(\sum_{t\in \left(20,\;30\right)}\frac{TP_{t}}{TP_{t}+FN_{t}}\right)$$

In Eq. (3), recall refers to the proportion of the segmented foreground that correlates with the ‘ground truth’ foreground.

The F1 score (also known as the balanced F-score or Dice coefficient) was then calculated as the harmonic mean of both precision and recall:

(4) $$F_{1}\;=\frac{1}{N}\left(\sum_{t\in \left(20,\;30\right)}\frac{2\;\times Precision_{t}\times Recall_{t}}{Precision_{t}+Recall_{t}}\right)$$

The F1 score can range between 0 – 1 and a lower result indicates greater deviation from the ‘ground truth’.

The False Positive Rate (FPR) was defined as:

(5) $$FPR=\frac{1}{N}\left(\sum_{t\in \left(20,\;30\right)}\frac{FP_{t}}{TN_{t}+FP_{t}}\right)$$

In Eq. (5), the FPR refers to the proportion of the negative ‘ground truth’ that is wrongly segmented as foreground.

In general, accuracy is used when the proportion of true results is the key issue, while the F1 score is employed when negative results are more important since it magnifies the effect of incorrect classification. While accuracy is an easy measure to understand, it has the drawback of giving a distorted representation of efficacy when there is a big imbalance in the number of negatives and positives. For face and head segmentation, missing portions of the face or addition of background features to the face are both undesirable, so the F1 score should be the preferred measure of segmentation efficacy.

For the three factors where no subject was present and the ‘ground truth’ consists of complete background, the preferred measure would be the FPR since all errors consist of false positives. For the ‘Ghost Images’ and ‘Dynamic Background’ factors, a low FPR indicates the algorithm has the ability to rapidly remove ghost images and cope with dynamic background objects respectively. However, for the ‘Sleeping Foreground’ factor, a low FPR indicates that the stationary foreground object has been absorbed into the background model and no longer shows up.

Segmentation efficacy should be calculated on a per-image basis to distinguish algorithms that vary in performance depending on image content, and ought to include a measure of spread in addition to centrality. This is especially important when evaluating algorithms that have a temporal component, as the segmentation result can change even though the images in the clip may be broadly similar.

### 2.4 Simulated factors

Gaussian and Uniform noise was added to the standard clip according to the method used by López-Rubio, with four levels of each type [11]. Jitter was simulated using a soft rubber mallet to strike the supporting tripod at regular intervals to induce vibrations. This was done to mimic both instability in the camera support as well as residual alignment differences that remain after motion compensation during pre-processing.

The two types of sudden illumination changes which are common in indoor settings come from switching overhead lights or drawing curtains (global change), and from opening and closing room doors which lead to an external light source (directional or local change). This was simulated by dimming and brightening all lights at once (global change), or by doing so with the lights from one direction only (local change). In contrast, the effect of shadows from uneven illumination was simulated by keeping the lights from one direction turned off throughout the clip duration. There was no temporal variance in the illumination, compared to the local change where the lighting was altered partway through the clip.

The requirement for background initialization was simulated by trimming the standard clip to show the subject in a stable stance (no initialization), or with two seconds of clear background (short initialization) at the start. In both cases, the comparison period was shifted to 10 s after the clip start to align with standard clip processing.

Simple color camouflage was simulated by using a cream-colored background screen, which was similar in color to the subject's skin. The subject also changed clothing to match the background color. This color was not exactly matched as it would be an unfair test since even humans would have difficulty differentiating the subject's outline under such conditions. For complex color camouflage, this was simulated by removing the background screen to show a typical indoor scene under daylight. The lighting for the subject was still controlled using the same bidirectional studio lights, and portions of the background were of similar color to the subject's clothing, skin, and hair.

For the final three factors, no subject was present and the ‘ground truth’ was set to complete background. Simulation of ‘Ghost Images’ was achieved by draping a complex patterned cloth over part of the background screen and dropping it at the 10 s mark. The comparison period was adjusted to start one second after the cloth completely left the image frame to test whether the algorithm could rapidly remove ‘Ghosts’.

Simulation of ‘Sleeping Foreground’ was done by lowering the same patterned cloth over part of the background screen at the 10 s mark. The comparison period was set to start 10 s after the cloth was completely lowered and had achieved a stable position. This was to assess the algorithm's tendency to absorb stationary foreground objects into the background. A ‘Dynamic Background’ was simulated using a running stand fan placed in the same position where the subject would normally stand. This was to test the algorithm's ability to cope with background objects that display regular repetitive motion.

### 2.5 Comparison results

The results for comparison testing of efficacy and processing speed for the algorithms in the ‘BGSLibrary’ were summarized in Table 3 and Table 4 respectively. The measure of segmentation efficacy used was the F1 score, except for the three factors without a foreground subject where the FPR was used instead. Efficacy was calculated on a per-frame basis and the results displayed in each cell were for the 10th centile, median, and 90th centile frames, thus showing both centrality and spread. Processing speed was calculated as the average for all frames during the comparison period.

### 2.6 Assessment criteria

The results were assessed according to the following criteria: 1) high efficacy to properly segment the head and upper body, 2) consistency, 3) processing speed short enough for real-time segmentation, and 4) tolerance to the factors tested (Table 5). Good segmentation is defined as a well-demarcated outline for the foreground object with at most small areas that are incorrectly classified but can be separated from the true foreground. Adequate segmentation is defined as a recognizable outline for the foreground object, with larger areas of incorrect classification that can still be separated from the foreground. From Fig. 2, it can be estimated that good segmentation corresponds to a F1 score of 0.95 and above, while adequate segmentation corresponds to a F1 score from 0.80 to 0.95.

To satisfy the criteria for high efficacy, the median F1 score should be 0.95 and above under standard processing conditions, while the segmentation should be consistent enough that the 10th centile F1 score does not go below 0.80. For full real-time processing with a frame rate of 30 fps, each frame should require a maximum of 33 ms. With a more relaxed constraint of 10 fps if the intervening frames can be motion interpolated, the segmentation time for each frame should be a maximum of 100 ms. An algorithm is considered to tolerant to the factor tested if it can still segment adequately with a median F1 score above 0.80.

To establish a cutoff for the ‘Ghost Images’ and ‘Sleeping Foreground’ factors, we need to refer to Table 3 where the maximum FPR is approximately 0.45, corresponding to the full coverage of the patterned cloth over the background. We can then assume that an FPR of less than 5% of the maximum value at the 90th centile (FPR = 0.020) indicates that the tested algorithm can rapidly deal with ‘Ghosts’ which are no longer visible during the whole comparison period. Similarly, an FPR less than 5% maximum at the 10th centile indicates that the static foreground object has started to integrate into the background during the comparison period.

For the ‘Dynamic Background’ factor, the highest median FPR is for the ‘Simple Gaussian’ and ‘Fuzzy Gaussian’ algorithms. On viewing both, the ‘Fuzzy Gaussian’ clip has less noise and the maximum FPR representing the area of the moving fan blades can be derived from integrating the segmented foreground over the comparison period of the clip and removing pixel noise with a small kernel median filter. This value was found to be 0.0563, and we can similarly assume that a median FPR less than 5% of this value (FPR = 0.003) indicates that the algorithm can adequately deal with dynamic backgrounds by removing the spinning fan blades.

### 2.7 Limitations

There are several limitations to the methodology of the comparison testing. The first is that the ‘ground truth’ could not be consistently derived from the depth data since the disparity maps were coarse and unstable, even after extensive pre-processing with temporal and spatial smoothing with hole-filling. While the use of automated GrabCut improved this significantly, there was still a need to manually inspect each frame for quality. Areas which were prone to incorrect segmentation had to be marked out and corrected manually (Fig. 1). This imposed a restriction on the amount of movement the subject could make, since excessive motion would require the markings to be updated every few frames instead of being allowed to propagate for much longer.

The next limitation is that the camera was static and true motion was not tested. This is important since many use cases require a mobile camera. However, this was unavoidable since the quality of the disparity maps would deteriorate even further with movement.

Another limitation is that while the ‘memory effect’ was demonstrated for several of the routines, it is possible that extending the test period may have detected it in more of them. Examination of the full clips for the routines which satisfied the stability criterion did indicate that towards the end of the clips there was deterioration in segmentation efficacy for some of them.

One more limitation is that there is only a single subject in all the video clips. While this does make the testing conditions consistent and improves the comparability of the results between factors, having more subjects with different combinations of hair and skin color will make the results more generalizable.

The final limitation is that only temporal segmentation routines were tested formally. It is possible that the non-temporal routines may eventually prove to be more suited to head and upper body segmentation. There is however no equivalent to the ‘BGSLibrary’ for non-temporal algorithms, and this is a gap in the current body of research.

### 2.8 Future directions

The first step would be to use a better depth camera to retake the test video clips, and the upcoming Intel RealSense L515 which uses solid state light detection and ranging (LiDAR) technology seems to be a big improvement on the D435 model used in this study. It has a depth error standard deviation of only 2.5 mm at 1 m distance from the target, gives cleaner contour outlines, has a higher resolution, and scans fast enough to cope with motion. This would remove the need for manual intervention when determining the ‘ground truth’ and would allow testing of both subject and camera motion.

Another step would be to gather and test non-temporal segmentation algorithms in a new library using the same methodology. Although it will require a lot of effort, this is necessary if we wish to identify suitable routines for head and upper body segmentation, since the routines from the ‘BGSLibrary’ are poorly suited for this.

The third step would be to expand the series of clips to cover background segmentation of the face only, since some use cases do not require the whole head and upper body. Examples of this would be face expression analysis and computation of facial action units.

## Ethics Statement

The only human subject was the first author for which informed consent was sought and obtained. The study has ethics approval from the University of Auckland Human Participants Ethics Committee (Ref. 021497).

## Funding

This study was paid for using PRESS account funding from the 10.13039/501100001537University of Auckland (ID 663710048).

## Declaration of Competing Interest

The authors declare that they have no known competing financial interests or personal relationships which have, or could be perceived to have, influenced the work reported in this article.
